# Family Income Affects Children’s Altruistic Behavior in the Dictator Game

**DOI:** 10.1371/journal.pone.0080419

**Published:** 2013-11-12

**Authors:** Yongxiang Chen, Liqi Zhu, Zhe Chen

**Affiliations:** 1 Key Laboratory of Behavioral Science, Institute of Psychology, Chinese Academy of Sciences, Beijing, China; 2 University of Chinese Academy of Sciences, Beijing, China; 3 Department of Human and Community Development, University of California, Davis, California, United States of America; University of Western Brittany, France

## Abstract

This study aimed to examine how family income and social distance influence young rural Chinese children’s altruistic behavior in the dictator game (DG). A total of 469 four-year-old children from eight rural areas in China, including many children left behind by parents who had migrated to urban areas for work, played the DG. Stickers comprised the resource, while recipients in the game were assumed to be either their friends or strangers, with the social distance (i.e., strangers compared to friends) as a between-subjects variable. Children donated significantly more stickers to their friends than to strangers. Moreover, children from lower income families donated more stickers than children from higher income families. However, no gender and parental migrant status differences in children’s prosocial behaviors were evident in this sample. Findings of this study suggest that children’s altruistic behaviours to peers are influenced by family characteristics since preschool age. The probable influence of local socialization practices on development and the possible adaptive significance were discussed.

## Introduction

Altruistic behaviors directed toward non-kin individuals have been rewarded across human cultures [1,2]. To explore the essence of altruism, researchers have focused on children to track their development in this area [3–5]. Recent studies have found that even young preschoolers behave altruistically in simple economic games, and their altruistic behavior increases consistently from preschool years to early school age [3–5]. Relatively few studies have investigated the factors in social context that influence young children’s altruism. Although previous studies revealed that age is most likely a factor that influences altruism (e.g., see[6,7]), examination of developmental patterns is beyond the scope of the present study. In this study, we addressed the issues of how socioeconomic status (SES) (which refers to family income in this study) and social distance (i.e., strangers compared to friends) influence preschoolers’ altruistic behaviors in the dictator game (DG), using a large sample of children from rural China.

The DG has been widely used to assess children’s and adults’ altruistic behavior in diverse cultures because of its simplicity, uniform procedure [4] and tightly controlled situation [8]. In a typical DG, the proposer (or dictator) is given an amount of money or some valued resource and asked to divide it between himself/herself and the recipient (or receiver). Both the proposer and the recipient are anonymous to each other; thus a rational proposer is assumed to be thoroughly pro-self and expected to give zero. Yet in previous studies, players from diverse cultures did donate a portion of the stake consistently, with the amount of the donation influenced by cultural and individual differences [8].

Though researchers have begun to address issues of how genetic and environmental factors interact to shape children’s prosocial behaviors [9], social contexts that shape children’s altruistic behaviors remain largely unexplored. In a classic study across 15 different cultures, Henrich and colleagues suggested that the degree of market integration and the payoffs of cooperation in everyday life would affect adults’ altruistic behavior to anonymous others, and that participants from more traditional societies would be most willing to share with others [1]. Other studies have addressed issues of cultural influences on children’s prosocial behaviors. For example, Rochat and Dias tested three- and five- year-olds in seven cultures, including a group of middle SES preschoolers (*N*=41) in urban Shanghai, China, distributing small numbers of desirable candies in 2009 [10]. All the Chinese children were recruited from “a large communist party–run preschool” in Shanghai, and the results showed that preschoolers in China were quite prosocial in the game. However, China is a large nation with a wide diversity. There are large and highly urbanized regions (e.g., booming cities such as Shanghai), and there are also vast rural areas in which many people are still struggling with poverty. Children from socioeconomic status (SES) environments thus may vary in their behavirors, as Benenson and colleagues found, children aged nine from higher socioeconomic status (SES) environments in England behaved more altruistically than those from lower SES contexts in the dictator game [4]. In Benenson’s study, a higher SES school was defined as one where less than 5% of children received free lunches; a lower SES school was one where more than 50% of children received free lunches. Benenson’s study was conducted in a western, developed country, yet, the economical environment varies a lot in China, as an eastern, developing country. For example, a specific phenomemon in China is that a large group of rural children were left behind when their parents migrated to cities to work. This practice has attracted sociological and psychological researchers’ attention since the last decade of the 20^th^ century [11]. 

According to a national survey in 2008, about 140 million farmers in rural China had migrated to cities to work and about 58 million children were left behind at their rural homes [12], staying with extended family members. These children are referred to as “left-behind children” [13]. Among all the left-behind children in rural China, about 27% are of preschool age; and most of them lived with their grandparents and/or one parent since being left behind [12]. It has been demonstrated that left-behind children of school age in rural China are disadvantaged in health behavior and school engagement, and children's psychosocial environment (including family SES and peer support) is associated with their developmental outcomes [13]. However, it is not clear whether children’s prosocial behaviors would be influenced by their early experiences of being left behind. 

Apart from the challenges of being left behind and/or being in poverty, many children in China have to face another special situation, as many of them are only children without any siblings due to China’s “one-child-per-family” policy , that has been in effect since the late 1970s [14]. Some researchers have been worried that only children might be spoilt by their family members and become self-centred “little emperors” [15] [16]. Other researchers have examined the social development of school age children and found that being only child is not associated with behavior problems [17]. It was further demonstrated that Chinese adolescent only children outperformed their counterparts who have siblings in terms of many psycho-behavioural characteristics, for example, being more involved in making donations to benevolent organizations [18]. However, whether young preschool children’s prosocial behaviors are influenced by their status of being only children or not remains largely unexplored. Thus it is of particular interest to investigate how the factors mentioned above could possibly influence children’s altruistic behaviors in China, as such a rapidly developing and changing society.

In addition to the SES and family structure variables, we are also interested in how young children would behave when playing DG with receivers of different social distance (i.e., strangers compared to friends). Previous studies have found that college students would give more to friends (not knowing which friend) than to a stranger in DG [19]. Also, dictators of school age would behave more altruistic in DG as the social distance between dictator and receiver decreases, they would donate most money to their closest friends [20]. In the present study, we examined whether social distance (i.e., strangers and friends) between dictator and receiver would influence younger children namely preschool children’s DG allocations. We further investigated how SES and family structure could possibly influence children’s allocations to receivers of different social distance in DG.

To explore the factors that influence children’s altruistic behavior, a large sample of four-year-old (3:6 to 4:6) children were included in this study. This age range was chosen as three- to four-year- olds enroll in preschool/kindergarten in most areas of China. All participants in the current study were recruited from local preschools/kindergartens, thus all children have some, though not necessarily comparable, experience interacting with both strangers and friends. The specific variables of interest were: whether preschoolers in China would exhibit altruistic behaviors differently towards friends and strangers in DG, whether such behaviors would be shaped by the social contexts in which they were raised [e.g., family income, number of siblings, number of parents having migrated]. To explore the influence of SES, we chose an individual level variable, that is, average family income per year per person, as reported by each child’s parent or caregiver.

We hypothesized that children of preschool age would donate more to friends than to strangers in DG. Furthermore, in the context of rural China, though quite different from the context in England (see [4]), we hypothesized that children’s altruistic behaviors in DG would be associated with their family income, and left-behind children would be different to non-left-behind children in their altruistic behaviors.

## Method

### Participants

This study was approved by the Institutional Review Board at Institute of Psychology, Chinese Academy of Sciences. Written informed consent was obtained from each participant’s parent or main care-giver (when both parents of the child migrated for work). Participants in the study consisted of 469 kindergarten children (252 boys and 217 girls) in eight counties in rural China. The sites were distributed evenly in four large areas in China, including two in northern China (Qingyuan and Wuyi in Hebei Province), two in central China (Jiangyong in Hunan Province and Luanchuan in Henan Province), two in eastern China (Yuexi and Huoqiu in Anhui Province), and two in southwest China (Renshou and Jingyan in Sichuan Province). The sample size from each site varied from 24 to 74 children, due to sampling convenience. The broad sample is moderately large as well as ethnically and economically diverse. More than 88% of the children were of Han nationality, the major ethnic group (over 90% of the population) in China. 

Assessments were conducted when the children were four years old, ranging in age from 3:6 to 4:6; all the children were recruited from local kindergartens. Children’s parents or primary caregivers reported sibling status and other demographic variables in questionnaires: 63.9% of children’s fathers were farmers. More than half (53.0%) of the children in the sample were only children, while the remainder had one or more than one sibling. 62.0% of participants had more than five family members, usually including the child’s parents and grandparents. In the present study, among all 251 migrant families, father-only migration is most common (135 families, 54%), followed by two-parent migration (79 families, 31%) and then mother-only migration (37 families, 15%). This pattern is slightly different from that in Wen’s study [13], in which two-parent migration was common. Presumably this was due to the fact that children in the present study were substantially younger than those in Wen’s study, and relatively few mothers migrated to work when children were still at their preschool age.

### Procedure

The paradigm was a modified version of the classic dictator game; there were no real receivers at the site. The scenario was a story-like situation that children usually experience in kindergartens. Social distance was a between-subject variable with the levels of stranger and friend. Children in each site were randomly assigned to either the stranger or friend condition. All children participated in the dictator game only once, as proposers. 

Children were interviewed one by one in a quiet room. They were told to play games, but were not told what games they were going to play before they entered the meeting room. So children in both conditions did not know what other children have done before the dictator game began. At the time of testing, they were separated from each other, and they did not know what other children (the possible responders, i.e., friends or strangers) were doing. 

Each game was played with an endowment of four stickers. Attractive stickers were used as resources. These stickers were selected as they are highly valued by children of this age in China. Kindergarten teachers usually use them to reward children’s good manners. We adjusted the number of stickers to four in DG to ensure that children’s performance would not be affected by their numerical knowledge. 

At the time of testing, each child was brought individually, from their classroom, to a quiet room by an interviewer. The child and the interviewer sat across from each other at a child-sized table. The interviewer first introduced herself and asked the child’s name. Then she informed the child that she had four stickers for the child, and she laid them out on the table, in front of the child. The interviewer then declared that the child would play a game with a partner. The partner was explained according to the condition in which the child participated.

In the friend condition, the interviewer would ask who the child’s best friend was. Most children named a child to this question, although a few children were not immediately sure. In these cases, the interviewer would ask the child who he/she often played with in the kindergarten. Once the child identified the best friend, the interviewer displayed a set of four stickers and declared that he/she would play a game with his/her best friend. 

Standard instruction for the friend condition was as follows: “Would you please tell me who is your best friend in your class? OK, now suppose you are going to play a distribution game with him/her. Now here are four stickers, they are all yours, you can distribute them between you and your friend. Your friend can only agree with you and receive whatever number you give to him/her (“Ta”, in Chinese); Ta cannot reject your offer. You may distribute one, two, three, or all four stickers to him/her, or you could give Ta nothing and keep them all. So, would you like to give any of those to your friend? How many of them would you like to give? You may give him/her one, two, three, or all four stickers, or you could keep all to yourself. It is up to you.” To make sure the child understood the number donated, the interviewer would count the stickers slowly when the child was watching. The stickers were given to children afterwards. 

In the stranger condition, the interviewer told the child that he/she (“Ta”, in Chinese) would play the game with a peer that Ta did not know (i.e., a stranger). The instruction for the stranger condition would begin as follows: “Now you are going to play a distribution game with a child at your age that you do not know. And Ta (meaning he or she) would not know who you are.” The procedure following this instruction was identical to that in the friend condition. 

## Results

The results of the present study were analyzed using SPSS 16.0. The data are stored in the Institute Lab and are accessible to researchers who are interested in it for academic reasons. Please write to the corresponding author, and we are happy to share the data.

**Figure 1:**
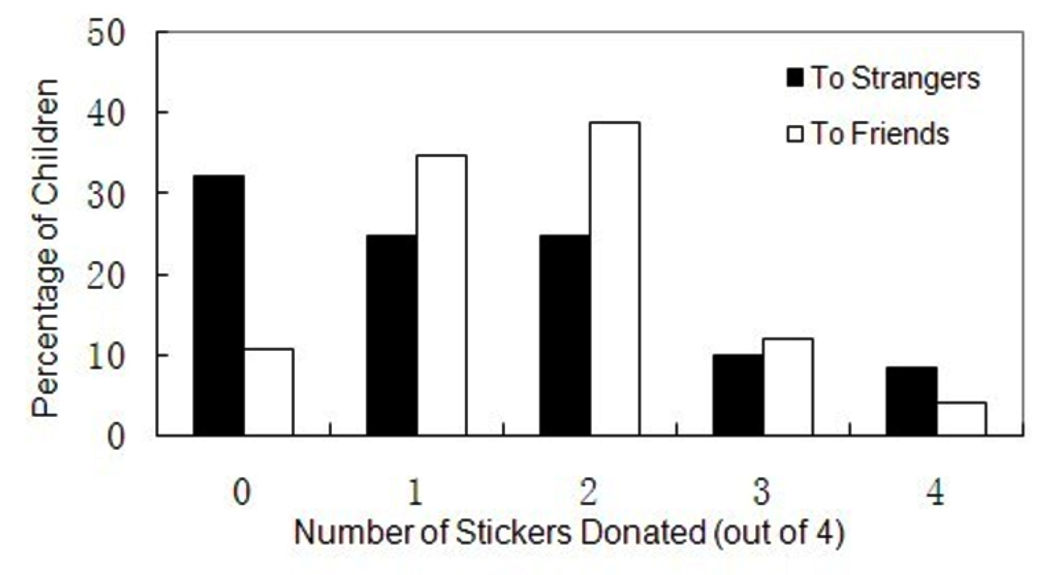
Distribution of number of stickers donated in DG, by social distance levels (between-subject), for the complete sample.

One-sample Kolmogorov-Smirnov tests were carried out to examine the distribution pattern of children’s DG offers. The results showed that children’s DG offers to friends (*M* = 1.64, *N* = 225, *p* = .065) and to strangers (*M* = 1.37, *N* = 230, *p* = .229) both followed Possion distributions (but not Normal distributions). The results of the Mann-Whitney U test showed significant differences between these two distributions (*p* = .002). As Figure 1 demonstrates, more participants would give nothing to strangers than to friends [strangers: 32.2%, *N* = 230; friends: 10.7%, *N* = 225; Pearson χ^2^(1) = 31.13, *p* = .005], and more participants chose equal distribution to friends than to strangers [strangers: 24.8%, *N* = 230; friends: 38.7%, *N* = 225; Pearson χ^2^(1) = 10.14, *p* = .005], though the contribution patterns of both social level conditions were basically distributed over the range [0, .5]. Moreover, 16.0% of the children in friend condition, and 18.3% of the children in stranger condition, gave more than half of the stickers to others (the difference was not significant, Pearson χ^2^(1) = 0.10, *p* = .750). This findings of children’s generosity are further discussed in the Discussion section.

As children in every sample site were randomly distributed to stranger or friend conditions, all the independent variables in Table 1 showed no significant differences between the two social distance levels. So the two groups were comparable as far as we mainly focused on the variables listed in Table 1. Among all participants, 16.8% of them were left-behind as both parents migrated, 28.7% were left-behind as only fathers migrated, 7.9% were left-behind as only mothers migrated, and 24.7% were non-left-behind children. 

**Table 1:** Descriptive statistics (mean±SD/percentage).

	No-parent migration	Mother-only migration	Father-only migration	Both-parent migration	*p*
Dependant variable					
Children’s DG offers	1.47 ± 1.19	1.36 ± 1.05	1.40 ± 1.04	1.69 ± 1.17	.355
DG offers to friends	1.47 ±. 97	1.50 ± .86	1.51 ± .94	1.97 ± 1.01	.077
DG offers to strangers	1.47 ± 1.35	1.22 ± 1.22	1.30 ± 1.14	1.39 ± 1.27	.938
Independent variables					
Children’s Age (years)	4.07 ± .31	4.02 ± .34	3.94 ± .29	3.98 ± .26	.017
Gender (male=1)	.56	.49	.56	.53	.852
Number of children in the family	1.54 ± .61	1.35 ± .54	1.52 ± .61	1.51 ± .63	.381
Maternal education (years)	9.35 ± 2.34	9.60 ± 2.46	9.38 ± 2.40	8.84 ± 1.53	.512
Paternal education (years)	9.95 ± 2.30	10.31 ± 2.70	9.77 ± 2.57	9.29 ± 1.62	.208
Family income (in 1000 RMB)	5.00 ± .34	5.16 ± .40	6.12 ± .61	5.46 ± .51	.975
Social distance (strangers=1)	.56	.51	.50	.47	.611

Note. Children’s DG offers = Children’s DG offers in the whole sample (*N* = 455). *N* = 225 for friend condition, *N* = 230 for stranger condition. Family income = Average annual per capita family income, median. one hundred Yuan RMB = 14.95 US dollars at the time of testing.

In order to examine whether parental migrant status affects family income and other variables, Kruskal-Wallis H tests were carried out, and the *p* values were listed in Table 1. As shown in Table 1, though left-behind children seem offer more stickers than the non-left-behind, the difference was not significant (*p* = .355). Family income did not differ significantly (*p* = .975) between left-behind and non-left-behind children either. Except children’s age (*p* = .017), all other variables listed in Table 1 did not differ significantly by parental migrant status. In the following part, we will further analyze how children’s age and other variables are related. 

The correlations of social distance, SES variables and DG offers are shown in Table 2. In the whole sample, we found that social distance (Spearman *r* = -.15, *p* = .002) and family income (Spearman *r* = -.10, *p* = .037) were negatively related to DG offers. Other demographic and SES variables were not significantly related to DG offers. Overall, children donated more stickers to friends than to strangers, and children from lower income families donated more than children from higher income families. Correlation analyses also showed that parental migrant status was related with children’s age (Spearman *r* = -.13, *p* = .015); when children were still at a young age, it was more likely that only fathers migrated to work and mothers stayed at home. Moreover, it should be noted that parental migrant status was not related to family income significantly (Spearman *r* = -.01, *p* = .848). 

**Table 2:** Spearman correlations of DG offers with child, family and SES variables.

	wDG	fDG	sDG	1	2	3	4	5	6	7	8	9
1 Social distance	-.15**	.	.									
2 Children’s Age	-0.03	-0.04	-0.04	-0.02								
3 Children’s Gender	-0.01	-0.02	-0.01	-0.03	-0.02							
4 Number of children	0.02	0.01	0.02	-0.03	0.05	0.01						
5 Mo-migration	-0.03	-0.03	-0.04	0	0.02	-0.04	-0.09					
6 Fa-migration	-0.04	-0.06	-0.02	-0.02	-.14**	0.02	0.01	-.26**				
7 Bo-migration	0.1	.20**	0.01	-0.05	-0.02	-0.02	0.01	-.18**	-.40**			
8 Maternal education	-0.07	-0.1	-0.05	0.01	-0.04	-0.04	-.31**	0.04	0.03	-0.08		
9 Paternal education	-0.01	-0.1	0.06	-0.05	0.08	-0.03	-.22**	0.06	-0.03	-0.09	.51**	
10 Family income	-.10*	-.19**	-0.03	-0.04	-0.09	-0.06	-.31**	0	0.02	-0.02	.25**	.18**

Note. wDG = Children’s DG offers in the whole sample (*N* = 455), fDG = DG offers to friends (*N* = 225), sDG = DG offers to strangers (*N* = 230). Social distance (0 = friends, 1 = strangers). Number of children= Number of children in the family. Family income = Average annual per capita family income. Mo-migration = Mother-only migration, Fa-migration = Father-only migration, Bo-migration = Both-parent migration.

* *p* < .05, **p < .01, ***p < .001, all two-tailed.

a Cannot be computed because at least one of the variables is constant.

In the friend condition, both-parent migration was positively related to DG offers (Spearman *r* = .20, *p* = .009), and family income was negatively related to DG offers (Spearman *r* = -.19, *p* = .005). It seems that children who were left behind by their migrant parents donated more to their friends than other children, and children from lower income families donated more to their friends than children from higher income families (see Table 2). However, none of the explanatory variables listed in Table 2 were significantly related to children’s DG offers to strangers. In the following section, we further examined possible interactions between social distance and other variables.

From the correlation analyses (see Table 2), three potential predictors (i.e., social distance, family income, and both-parent migration) of DG offers were found. We further explored whether there were any interactions among them, using hierarchical regression analyses. The continuous predictors were centered, represented as deviations from their own (sample) means. As shown in Table 3, the three potential predictors were entered in the first step. In the second step, the two-way interactions (i.e., social distance * family income, family income * both-parent migration, and social distance * both-parent migration) were entered. In the third step, the three-way interaction (i.e., social distance * family income * both-parent migration) was entered. Beta coefficients (*β* ) and *t* values (at entry as well as in the final equation) are reported for each variable, and variance accounted for (*R* ^*2*^) and change in *R* ^*2*^ are reported for each step in Table 3. For the whole sample, no significant interaction term was found. Therefore, it seemed that family income did not affect young children's giving to friends and strangers, and parental migrant status did not influence children’s prosocial behaviors in DG in this sample.

**Table 3:** Examination of Possible Interactions among Three Independent Variables.

Step Predictors	*t*	*p*	*β*	*R* ^*2*^	△*R*2	*F* change
*Children’s DG offers*						
1 Social distance	-1.825	.069	-.098	.031	.031	3.581*
Family income	-2.024	.044	-.109			
BPM	1.819	.070	.098			
2 Social distance	-1.112	.267	-.067	.040	.009	1.028
Family income	-2.217	.027	-.172			
BPM	2.137	.033	.161			
Social distance * Family income	1.280	.201	.091			
Family income * BPM	.066	.947	.004			
Social distance * BPM	-1.151	.250	-.092			
3 Social distance	-1.111	.267	-.067	.040	.000	.007
Family income	-2.137	.033	-.174			
BPM	2.129	.034	.161			
Social distance * Family income	1.180	.239	.094			
Family income * BPM	.102	.919	.008			
Social distance * BPM	-1.152	.250	-.092			
Social distance * Family income * BPM	-.081	.935	-.006			

Note. Social distance (0 = friends, 1 = strangers). Family Income = Average annual per capita family income (1000 RMB). BPM = Both-parent migration.

* *p* < .05, **p < .01, all two-tailed.

Since no interaction term was significant, we refitted the model without the insignificant interactions so that other effects might be better assessed. Hierarchical regression analyses were used to examine the influence of social distance and family income, and to determine how much variance they accounted for. Beta coefficients (*β* ) and *t* values (at entry as well as in the final equation) are reported for each variable, and variance accounted for (*R* ^*2*^) and change in *R* ^*2*^ are reported for each step (see Table 4).

**Table 4:** Regressions predicting DG offers.

Step Predictors	*t*	*β*	*R* ^*2*^	△*R*2	*F* change
*Children’s DG offers*					
1 Social distance	-2.14^*^	-.10	.01	.01	4.56^*^
2 Social distance	-2.28^*^	-.11			
Family Income	-2.75^**^	-.13	.03	.02	7.54^**^

Note. Social distance (0 = friends, 1 = strangers). Family Income = Average annual per capita family income (1000 RMB).

* *p* < .05, **p < .01, all two-tailed.

The regression analyses showed that both predictors significantly contributed to the DG offers. These results revealed that children from lower-income families were more altruistic than those from higher-income families (β = -.13, *p* = .006), and children donated more to their friends than to strangers (β = -.11, *p* =.023). Some of our hypotheses were confirmed by the regression analyses, as it is evident that both social distance and family income significantly contributed to whole DG offers in preschool children. However, the relationship between parental migrant status and children’s prosocial behaviors was not significant. Moreover, it should be noted that family income and social distance only explained a small portion of variances. We will discuss this in the following session. 

## Discussion

The current study contributes to our understanding of factors impacting children’s altruistic behaviors, especially among rural Chinese preschool children. First of all, the results of this study show that children’s altruism decreased with increasing family income. The results are consistent with studies with adults that show that adversities increase people’s prosocial behaviors [21,22]. However, the results differ from Benenson’s study [4] conducted in England, which showed that SES does not affect children’s altruism in DG until they reach nine years old, when children’s mean DG offer increases with increasing SES levels.

Why does SES seem to exert different impacts on English and Chinese children’s altruism? Children in Benenson’s study [4] played DG with only strangers, and the results showed that children’s altruisitic behaviors in DG were not influenced by SES until children reached school age. In the present study, consistent with Benenson’s (2007) study, preschoolers’ altruism to strangers was not influenced by SES [4], according to the correlation analyses. However, young children’s DG allocations to friends was related to SES in preschool age in our sample. These findings thus suggest that the mechanisms underlying human being’s prosocial behaviors to friends and to strangers may differ since preschool age (or even earlier). Children as young as four years old might have more chances to observe adults’ interations with friends than to observe people’s interactions with strangers, and they have more chances to interact with friends than strangers in their daily life. This possibility may partially explain why children’s altruistic behaviors to strangers were not influenced by social context examined in the present study, while their DG allocations to friends varied with family income and their family migrant status. However, it remains unclear whether such a difference is inherent, or is caused by different early experiences, and how young children’s altruisitic behaviors are shaped by these two factors.

Another possible explanation for the discrepancy discussed above may involve cultural differences. According to Engel’s meta analysis and other cross-cultural studies [10], people from diverse cultures behave differently in DG. Benenson [4] examined the impact of SES differences in a western, developed country, while we did so in the context of a developing country that values collectivism. In western developed countries, impoverished families might receive help from social welfare/security. For instance, British children from lower SES families were eligible to receive free lunches in school, as reported in Benenson’s study [4]. However, in the present study, about one third of all children are raised in somewhat “absolutely impoverished” families. In a society that values collectivism, especially when people are in poverty or experiencing other adversities [22], they might have to rely on each other in their local society (e.g., their family members, relatives and friends) to get through hard days; thus reciprocal altruism [23,24] could be a sensible explanation in this case. 

Secondly, we found that young children’s prosocial behaviors were influenced by social distance. Some reseachers argue that human beings consider a small number of kin and friends as comprising the “we group” ( i.e., a specific kind of “ingroup”) and show an inherent preference for the members of that group in decision making (e.g., [25–27]), a phenomenon referred to as a kith-and-kin rationality. Previous study found that college students were more altruistic to people who were closer to them in terms of social distance[28]. The results of the present study demonstrate that kith-and-kin rationality may appear early in preschool years, as they donated more stickers to friends than to strangers. However, this result contradicted with another study which found that 5-8-year-old East Asian children in Canada seemed to show outgroup favoritism [29]. Here we should note that those children were Minority in Canada, and being prosocial to out-groups might help them adapt to local society. But children in the present study were “mainstream” themselves, and being prosocial to their friends might help them adapt to local community.

Although being left-behind is usually considered to be disadvantageous for children in China [13] as well as in other countries [30], it is demonstrated in the present study that simply being left-behind did not affect young children’s prosocial behaviors significantly. Children’s prosocial behaviors may be influenced by their early experiences interacting with their main care-givers (parents or grandparents, or other extended family members who take care of them when parents migrate)[31,32]. It may be a fruitful avenue for further investigation of how altruistic values of children’s caregivers and parental migrant status would influence older children’s prosocial behaviors. 

As noticed earlier, about 15-20% of the children in the present study would give more than half stickers (i.e., hyper-fair DG offers) to others in the dictator game. The hyper-fair children also exist in other studies using the dictator game (see [8]), however, the proportion of them seemed higher in the present study than that reported in previous studies (see Figure 10 in [8]), which were primarily carried out in western countries. A previous cross-cultural study showed that Chinese preschoolers were more likely to share resources with peers than Indian preschoolers, and the authors emphasized “the importance of cultural beliefs on young children’s behavior” (p. 219) [33]. Indeed, Chinese children are often told by parents and teachers to be modest, and to give valued resources to others; this has been deemed as a virtue in Chinese culture. For example, almost all children in China have heard the well known story that Kong Rong, a four-year-old boy in ancient China, gave the biggest pears to his brothers and only kept the smallest one to himself. This story is told in almost all kindergartens in China. The cultural influence may thus partly explain why some children were hyper-fair in dictator games in the present study. 

The present results did not show any effect of sibling interactions on prosocial behaviors to friends nor to strangers. Children with and without siblings did not differ in their prosocial behaviors to their friends or strangers. As about half children in the present study were only children in their families and the other half had siblings, we suggested that the absence of siblings might not affect rural preschool children’s prosocial behaviors to peers in China. This result is consistent with previous studies showing that behavior problems [17] and social values [34] in only children and children with siblings do not differ significantly. Moreover, children who grow up in rural communities probably spend a lot of time outdoors, thus social interaction opportunities of only children and children with siblings may not differ significantly. This may partly explain why they did not differ in terms of prosocial behaviors to friends and to strangers. 

As Engel pointed out in his meta analyses [8], researchers have tested gender effects in DG, the results showing that females give more and receive more resources in DG. However, gender differences are not evident in children’s prosocial behaviors in DG in the present study. This result was not consistent with those evidenced in the Fehr et al. study [5], which showed that boys demonstrated a stronger tendency of egalitarianism than girls. On the other hand, the present results contradict those in Andreoni & Vesterlund’s study [35] with adults, which found that females tend to prefer equity more than males. It is remains unclear whether the discrepancy between the present findings and those of the previous study (e.g., [35]) is due to cultural or developmental differences. 

Possible adaptive significance of children’s altruistic behaviors could be explained by social distance and family characteristics. Children from lower income families tended to be more altruistic than children from higher income families, this might help them get more resources through reciprocity [23,24] among people in their local community. Previous study showed that children of preschool age could likely expect more reciprocity from friends than from non-friends [36], and they might rely on close friends or relatives more heavily than strangers to conquer adversities that were accompanied with poverty. Generally, this is more or less consistent with the previous finding that adversities increase people’s prosocial behaviors in Chinese adults [22]. The results of the present study imply that children as young as four years old might have adopted some of adults’ preferences to guide their altuistic behaviors. This might help them to adapt to local society. 

One limitation of the present study was that the effects of family income and social distance were quite small, as shown in the regression analysis. However, it should also be noted that the participants in the present study were young preschool children, and they had limited social experiences. This might be a possible reason why family income and social distance only had small effects. In a previous study, 4-9 years old urban Chinese children were asked to donate stickers to either friends or strangers in dictator games, the results showed that in-group bias increased with children’s age[37]. Meanwhile, it is worth noting that some other variables (such as gender and parental migrant status) were not significant at all. This perhaps makes the small effects even more salient. It would be quite interesting if future studies could explore whether the effects of social distance and family income would change as children’s age increases in rural sample.

The evolution and mechanisms of altruism are hotly debated by researchers from different areas [8,38–40]. The current study suggests that culture and social context should be considered when examining how SES influences children’s altruistic behaviors. The present findings confirm that altruism emerges early in human early childhood [3,4], and possibly coevolved with culture groups [41]. However, the ecological, biological and cognitive mechanism of children’s altruistic behaviors remain largely unknown, as researchers point out, “altruism researchers must cooperate” (p. 653) in the future [40].
